# Supplementary material for: Stacking Model‐Based Classifiers for Dealing With Multiple Sets of Noisy Labels
Source: Biom J. 2025 Mar 12;67(2):e70042. doi: 10.1002/bimj.70042 (PMC11898607; doi:10.1002/bimj.70042)
Supplement: Supplementary file 1 — Supporting Information [file BIMJ-67-e70042-s001.zip › density-based-ensemble-model-main/competitors_real_data/netlab-master/netlab-master/help/demmlp2.htm]

Netlab Reference Manual demmlp2


# demmlp2

## Purpose

Demonstrate simple classification using a multi-layer perceptron

## Synopsis

```
demmlp2
```

## Description

The problem consists of input data in two dimensions drawn from a mixture
of three Gaussians: two of which are assigned to a single class. An MLP
with logistic outputs trained with a quasi-Newton optimisation algorithm is
compared with the optimal Bayesian decision rule.

## See Also

`mlp`, `mlpfwd`, `neterr`, `quasinew`

---

**Pages:**
Index


---

Copyright (c) Ian T Nabney (1996-9)
